# Controlling the Outbreak of COVID-19: A Noncooperative Game Perspective

**DOI:** 10.1109/ACCESS.2020.3040821

**Published:** 2020-11-26

**Authors:** Anupam Kumar Bairagi, Mehedi Masud, Do Hyeon Kim, Md. Shirajum Munir, Abdullah-Al Nahid, Sarder Fakhrul Abedin, Kazi Masudul Alam, Sujit Biswas, Sultan S. Alshamrani, Zhu Han, Choong Seon Hong

**Affiliations:** 1 Department of Computer Science and EngineeringKyung Hee University 26723 Yongin 17104 South Korea; 2 Department of Computer Science and EngineeringKhulna University 247289 Khulna 9208 Bangladesh; 3 Department of Computer ScienceCollege of Computers and Information TechnologyTaif University 125895 Taif 21944 Saudi Arabia; 4 Department of Electronics and Communication EngineeringKhulna University 247289 Khulna 9208 Bangladesh; 5 Department of Information Systems and TechnologyMid Sweden University 6311 851 70 Sundsvall Sweden; 6 Department of Computer Science and EngineeringFaridpur Engineering College Faridpur 7800 Bangladesh; 7 Department of Information TechnologyTaif University 125895 Taif 21944 Saudi Arabia; 8 Department of Electrical and Computer EngineeringUniversity of Houston 14743 Houston TX 77004 USA

**Keywords:** COVID-19, health economics, isolation, social distancing, noncooperative game, Nash equilibrium

## Abstract

*COVID-19* is a global epidemic. Till now, there is no remedy for this epidemic. However, isolation and social distancing are seemed to be effective preventive measures to control this pandemic. Therefore, in this article, an optimization problem is formulated that accommodates both isolation and social distancing features of the individuals. To promote social distancing, we solve the formulated problem by applying a noncooperative game that can provide an incentive for maintaining social distancing to prevent the spread of COVID-19. Furthermore, the sustainability of the lockdown policy is interpreted with the help of our proposed game-theoretic incentive model for maintaining social distancing where there exists a Nash equilibrium. Finally, we perform an extensive numerical analysis that shows the effectiveness of the proposed approach in terms of achieving the desired social-distancing to prevent the outbreak of the COVID-19 in a noncooperative environment. Numerical results show that the individual incentive increases more than 85% with an increasing percentage of home isolation from 25% to 100% for all considered scenarios. The numerical results also demonstrate that in a particular percentage of home isolation, the individual incentive decreases with an increasing number of individuals.

## I. Introduction

The novel Coronavirus (2019-nCoV or COVID-19) is considered to be one of the most dangerous pandemics of this century. COVID-19 has already affected every aspect of individual’s life i.e. politics, sovereignty, economy, education, religion, entertainment, sports, tourism, transportation, and manufacturing. It was first identified in Wuhan City, China on December 29, 2019, and within a short span of time, it spread out worldwide [1], [2]. The World Health Organization (WHO) has announced the COVID-19 outbreak as a Public Health Emergency of International Concern (PHEIC) and identified it as an epidemic on January 30, 2020 [3]. Till July 23, 2020, COVID-19 has affected 215 countries and territories throughout the globe and 2 international conveyances [4].

The recent statistics on COVID-19 also indicate that more than 15, 371, 829 persons have been affected in different ways [4], [5]. Currently, the ten most infected countries are USA, Brazil, India, Russia, South Africa, Peru, Mexico, Chile, Spain, UK, and these countries contributed more than 68% of worldwide cases. Since the outbreak, the total number of human death and recovery to/from COVID-19 are 630, 138 and 9, 348, 761, respectively [4], [5] (till July 23, 2020). The fatality of human life due to COVID-19 is frightening in numerous countries. For instance, among the highest mortality rates countries, 70% of the mortality belongs to the top 8 countries due to COVID-19. Furthermore, the percentages of affected cases for male and female are around 55.21% and 44.79%, whereas these values are about 76% and 24%, respectively in death cases globally [6]. Different countries are undertaking different initiatives to reduce the impact of the COVID-19 epidemic, but there is no clear-cut solution to date.

One of the most crucial tasks that countries need to do for understanding and controlling the spread of COVID-19 is testing. Testing allows infected bodies to acknowledge that they are already affected. This can be helpful for taking care of them, and also to decrease the possibility of contaminating others. In addition, testing is also essential for a proper response to the pandemic. It allows carrying evidence-based steps to slow down the spread of COVID-19. However, to date, the testing capability for COVID-19 is quite inadequate in most countries around the world. South Korea was the second COVID-19 infectious country after China during February 2020. However, mass testing may be one of the reasons why it succeeded to diminish the number of new infections in the first wave of the outbreak since it facilitates a rapid identification of potential outbreaks [7]. For detecting COVID-19, two kinds of tests are clinically carried out: (i) detection of virus particles in swabs collected from the mouth or nose, and (ii) estimating the antibody response to the virus in blood serum.

This COVID-19 epidemic is still uncontrolled in most countries. As a result, day by day, the infected cases and death graph are rising exponentially. However, researchers are also focusing on the learning-based mechanism for detecting COVID-19 infections [8]–[14]. This approach can be cost-effective and also possibly will take less time to perform the test. Some other studies [15]–[23] focus on finding the spreading behavior of COVID-19 by using known epidemic models like Susceptible - Infectious - Recovered (SIR), Susceptible - Infectious - Recovered - Susceptible (SIRS), Susceptible - Exposed - Infectious - Recovered (SEIR), Susceptible-Infected-Hibernator-Removed (SIHR), Susceptibl- Infected-Diagnosed-Ailing-Recognized-Threatened-Healed-Extinct (SIDARTHE) etc. However, all of these epidemic models are confined by the hypothesis of constant recovery rates, and they also struggle to reveal the system dynamics when there is a limited coupling between subpopulations [24]. Moreover, These models are inadequate to capture typically stochastic aspects, like fade-out, extinction, and lack of synchrony due to arbitrary delays [25]. Besides, the limitations of these models, they may present the epidemic scenario but have little impact on reducing or controlling the causes. However, the infected cases of the COVID-19 can be reduced by maintaining a certain social distance among the individuals. In particular, to maintain such social distancing, self-isolation, and community lockdown can be possible approaches. Thus, it is imperative to develop a model so that the social community can take a certain decision for self-isolation/lockdown to prevent the spread of COVID-19.

To the best of our knowledge, there is no study that focuses on the mathematical model for monitoring and controlling individual in a community setting to prevent this COVID-19 epidemic. Thus, the main contribution of this article is to develop an effective mathematical model with the help of global positioning system (GPS) information to fight against COVID-19 epidemic by monitoring and controlling individual. To this end, we make the following key contributions:

• First, we formulate an optimization problem for maximizing the social utility of individual considering both isolation and social distancing. Here, the optimization parameters are the positions of individual.
• Second, we reformulate the objective function which is incorporated with the social distancing feature of an individual as a noncooperative game. Here, we show that home isolation is the dominant strategy for all the individuals (players) of the game. We also prove that the game has a Nash Equilibrium (NE).
• Third, we interpret the sustainability of lockdown policy with the help of our model.
• Finally, we evaluate the effectiveness of the proposed approach with the help of extensive numerical analysis.

The remainder of this article is organized as follows. In Section II, we present the literature review. We explain the system model and present the problem formulation in Section III. The proposed solution approach of the above-mentioned problem is addressed in Section IV. We interpret the sustainability of lockdown policy with our model in Section V. In Section VI, we provide numerical analysis for the proposed approach. We present the limitation of the current study in Section VII. Finally, we draw some conclusions in Section VIII.

## II. Literature Review

COVID-19 is the seventh coronavirus identified to contaminate humans. Individuals were first affected by the 2019-nCoV virus from bats and other animals that were sold at the seafood market in Wuhan [26], [27]. Afterward, it began to spread from human to human mainly through respiratory droplets produced while individual sneeze cough or exhaling [3].

In [15], the authors present a generalized fractional-order SEIR model (SEIQRP) for predicting the potential outbreak of contagious diseases alike COVID-19. They also present a modified SEIQRP model in their work, namely the SEIQRPD model. With the real data of COVID-19, they have shown that the proposed model has a more reliable prediction capability for the succeeding two weeks. In [16], the authors introduce a Bayesian Heterogeneity Learning approach for Susceptible-Infected-Removal-Susceptible (SIRS) model. They formulate the SIRS model into a hierarchical structure and assign the Mixture of Finite mixtures priors for heterogeneity learning. They utilize the methodology to investigate the state level COVID-19 data in the U.S.A. The authors induce an innovative neurodynamical model of epidemics called Neuro-SIR in [17]. The proposed approach allows the modeling of pandemic processes in profoundly different populations and contagiousness contexts. In [18], the authors propose a mobility-based SIR model for epidemics considering the pandemic situations like COVID-19. The proposed model considers the population distribution and connectivity of different geographic locations across the globe. The authors propose a noble mathematical model for presenting the COVID-19 pandemic by fractional-order SIDARTHE model in [19]. They prove the existence of a steady solution of the fractional-order COVID-19 SIDARTHE model. They also produce the necessary conditions for the fractional order of four proposed control strategies. In [20], the authors propose a conceptual mathematical model for the epidemic dynamics using four compartments, namely Susceptible, Infected, Hospitalized, and Recovered. They investigate the stability of the equilibrium for the model using the basic reproduction number for knowing the austerity. In [21], the authors develop a pandemic model to inquire about the transmission dynamics of the COVID-19. Here, they assess the theoretical impact of probable control invasions like home quarantine, social distancing, cautious behavior, and other self-imposed measures. They apply the Bayesian approach and authorized data to figure out some of the model parameters. In [22], the authors introduce a SIHR model to prognosticate the course of the epidemic for finding an effective control scheme. The model parameters are estimated based on fitting to the published data of Hubei province, China. In [23], the authors present a mathematical model for COVID-19 based on three different compartments, namely susceptible, infected, and recovered classes. They also present some qualitative viewpoints for the model, i.e., the existence of equilibrium and its stability issues.

Machine learning can play an important role to detect COVID-19 infected individual based on the observatory data. The work in [8] proposes an algorithm to investigate the readings from the smartphone’s sensors to find the COVID 19 symptoms of a patient. Some commons symptoms of COVID-19 victims like fever, fatigue, headache, nausea, dry cough, lung CT imaging features, and shortness of breath can be captured by using the smartphone. This detection approach for COVID-19 is faster than the clinical diagnosis methods. The authors in [9] propose an artificial intelligence (AI) framework for obtaining the travel history of individual using a phone-based survey to classify them as no-risk, minimal-risk, moderate-risk, and high-risk of being affected with COVID-19. The model needs to be trained with the COVID-19 infected information of the areas where s/he visited to accurately predict the risk level of COVID-19. In [10], the authors develop a deep learning-based method (COVNet) to identify COVID -19 from the volumetric chest CT image. For measuring the accuracy of their system, they utilize community-acquired pneumonia (CAP) and other non-pneumonia CT images. The authors in [11] also use deep learning techniques for distinguishing COVID-19 pneumonia from Influenza-A viral pneumonia and healthy cases based on the pulmonary CT images. They use a location-attention classification model to categorize the images into the above three groups. Depth cameras and deep learning are applied to recognize unusual respiratory pattern of personnel remotely and accurately in [12]. They propose a novel and effective respiratory simulation model based on the characteristics of original respiratory signals. This model intends to fill the gap between large training datasets and infrequent real-world data. Multiple retrospective experiments were demonstrated to examine the performance of the system in the detection of speculated COVID-19 thoracic CT characteristics in [13]. A 3D volume review, namely “Corona score” is employed to assess the evolution of the disease in each victim over time. In [14], the authors use a pre-trained UNet to fragment the lung region for automatic detection of COVID-19 from a chest CT image. Afterward, they use a 3D deep neural network to estimate the probability of COVID-19 infections over the segmented 3D lung region. Their algorithm uses 499 CT volumes as a training dataset and 131 CT volumes as a test dataset and achieves 0.959 ROC AUC and 0.976 PR AUC. The study in [28] presents evidence of the diversity of human coronavirus, the rapid evolution of COVID-19, and their clinical and Epidemiological characteristics. The authors also develop a deep learning model for identifying COVID-19. and trained the model using a small CT image datasets. They find an accuracy of around 90% using a small CT image dataset.

In [29], the authors propose a stochastic transmission model for capturing the phenomenon of the COVID-19 outbreak by applying a new model to quantify the effectiveness of association tracing and isolation of cases at controlling a severe acute respiratory syndrome coronavirus 2 (SARS-CoV-2)-like pathogen. In their model, they analyze synopses with a varying number of initial cases, the basic reproduction number, the delay from symptom onset to isolation, the probability that contacts were traced, the proportion of transmission that occurred before symptom start, and the proportion of subclinical infections. They find that contact tracing and case isolation are capable enough to restrain a new outbreak of COVID-19 within 3 months. In [30], the authors present a risk-sensitive social distance recommendation system to ensure private safety from COVID-19. They formulate a social distance recommendation problem by characterizing Conditional Value-at-Risk (CVaR) for a personal area network (PAN) via Bluetooth beacon. They solve the formulated problem by proposing a two phases algorithm based on a linear normal model. In [31], the authors mainly dissect the various technological interventions made in the direction of COVID-19 impact management. Primarily, they focus on the use of emerging technologies such as Internet of Things (IoT), drones, artificial intelligence (AI), blockchain, and 5G in mitigating the impact of the COVID-19 pandemic.

Moreover, noncooperative game theory is used by different authors for solving resource allocation problems in communication [41], [42]. In [41], the authors represent the resource allocation problem as a noncooperative game, where every player desires to maximize its energy efficiency (EE). In [42], the authors modeled the distributed resource allocation problem as a noncooperative game in which every player optimizes its EE individually with the support of distributed remote radio heads.

The works [8]–[20], [28]–[31] focused on COVID-19 detection and analyzed the characteristic of its respiratory pattern. Hence, the literature has achieved a significant result in terms of post responses. In fact, it is also imperative to control the epidemic of COVID-19 by maintaining social distance. Therefore, different from the existing literature, we focus on the design of a model that can measure individual’s isolation and social distance to prevent the epidemic of COVID-19. The model considers both isolation and social distancing features of individuals to control the outbreak of COVID-19.

## III. System Model and Problem Formulation

**FIGURE 1.**
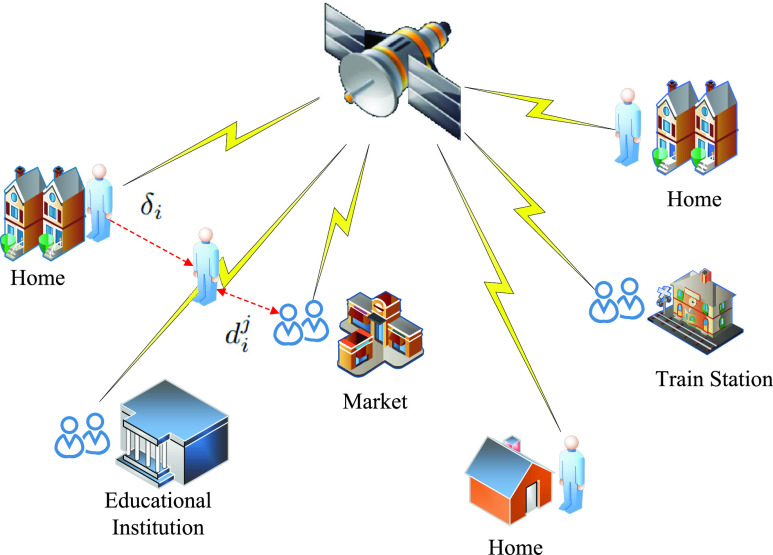
Exemplary System model. Isolation indicates staying at home whereas social distancing measures the distance of a individual from others.

Consider an area in which a set $\mathcal {N}$ of $N$ individuals are living under COVID-19 threat and must decide whether to stay at home or go leave their homes to visit a market, shop, train station, or other locations, as shown in Figure 1. Everyone has a mobile phone with GPS. From analyzing the GPS information, we can know their home locations of each individuals, and longitude and latitude of these locations are denoted by $\boldsymbol {X}^{h}$, and $\boldsymbol {Y}^{h}$, respectively. We consider one time period (e.g., 15 or 30 minutes) for our scenario and this time period is divided into $T$ smaller time steps in a set $\mathcal {T}$. For each of time step $t\in \mathcal {T}$, we have the GPS coordinates $\boldsymbol {X}$ and $\boldsymbol {Y}$ of every individual.

Now, the deviation from home for any individual $i\in \mathcal {N}$ in between two time steps can be measured by using Euclidean distance as follows:

\begin{align*} \delta _{i}^{t}= \begin{cases} \sqrt {(X_{i}^{h}-X_{i}^{t})^{2}+(Y_{i}^{h}-Y_{i}^{t})^{2}}, & \text {if}~t=1, \\ \sqrt {(X_{i}^{t-1}-X_{i}^{t})^{2}+(Y_{i}^{t-1}-Y_{i}^{t})^{2}}, & \text {otherwise}. \end{cases}\tag{1}\end{align*}

Thus, the total deviation from home by each individual $i\in \mathcal {N}$ in a particular time period can be calculated as follows:

\begin{equation*} \delta _{i}= \sum _{t\in \mathcal {T}}\delta _{i}^{t}, \forall i\in \mathcal {N}\tag{2}\end{equation*}

On the other hand, at the end of a particular time period, the distance between an individual $i\in \mathcal {N}$ and any other individuals $j\in \mathcal {N}, j\ne i$ is as follows:

\begin{equation*} d_{i}^{j}=\sqrt {(X_{i}^{T}-X_{j}^{T})^{2}+(Y_{i}^{T}-Y_{j}^{T})^{2}}.\tag{3}\end{equation*}

Hence, the total distance of individual $i\in \mathcal {N}$ from other individuals $\mathcal {N}_{i}\subseteq \mathcal {N}$, who are in close proximity with $i\in \mathcal {N}$, and $\mathcal {N}_{i}$ is fixed for a particular time step, can be expressed as follows:

\begin{equation*} d_{i}= \sum _{j\in \mathcal {N}_{i}}d_{i}^{j},\quad \forall i\in \mathcal {N}.\tag{4}\end{equation*}

Our objective is to keep $\delta $ minimum for reducing the spread of COVID-19 from infected individuals, which is an isolation strategy. Meanwhile, we want to maximize social distancing which mathematically translates into maximizing $d$ for reducing the chance of infection from others. However, we can use log term to bring fairness [32], [33] in the objective function among all individuals. Hence, we can pose the following optimization problem:

\begin{align*} &{\underset{\boldsymbol {X},\boldsymbol {Y}}{\max} } ~{\underset{i\in \mathcal {N}}{\min} } ~{\log (Z-\delta _{i})^\omega d_{i}^{(1-\omega)}} \tag{5}\\ &\text {s.t.} ~ \delta _{i}\le \delta _{\textrm {max}}, \tag{5a}\\ &\hphantom {\text {s.t.} ~} d_{i}^{j}\ge d_{\textrm {min}}, \quad \forall i,j \tag{5b}\\ &\hphantom {\text {s.t.} ~} \omega \in [{0,1}].\tag{5c} \end{align*}

In (5), $Z$ is a large number for changing the minimization problem to maximization one, and $Z>\delta _{i}, \forall i\in \mathcal {N}$. The optimization variables $\boldsymbol {X}$ and $\boldsymbol {Y}$ indicate longitude, and latitude, respectively, of the individuals. Moreover, the first term in (5) encourages individual for *isolation* whereas the second term in (5) encourages individual to maintain fair *social distancing*. In this way, solving (5) can play a vital role in our understanding on how to control the spread of COVID-19 among vast population in the society. Constraint (5a) guarantees small deviation to maintain emergency needs, while Constraint (5b) assures a minimum fair distance among all the individuals to reduce the spreading of COVID-19 from one individual to another. Usually, local government or authority can set the value of $\delta _{\textrm {max}}$, and $d_{min}$ can be set up by expertise body like the World Health Organization (WHO). Constraint (5c) shows that $\omega $ can take any value between 0 and 1 which captures the importance between two key factors captured in the objective function of (5). For example, if COVID-19 is already spreading in a given society, then most of the weight would go to isolation term rather than social distancing. Here, we present a utility-based model depending on the preventing mechanism like home isolation and social distancing. This is an indirect approach to combat an epidemic like COVID-19. We have no scope to combine traditional probabilities as described in epidemic models (i.e., SIR, SIRS, SEIR, SIHR, SIDARTHE, etc.) like infection and recovery in our utility-based model. The objective of (5) is difficult to achieve as it requires the involvement and coordination among all the $N$ individual. Moreover, if the individuals are not convinced then it is also difficult for the government to attain the objective forcefully. Thus, we need an alternative solution approach that encourage individual separately to achieve the objective and game theory, which is successfully used in [34], [35], can be one potential solution, which will be elaborated in the next section.

## IV. Noncooperative Game Solution

To attain the objective for a vast population, governments can introduce incentives for isolation and also for social distancing. Then every individual wants to maximize their utilities or payoffs. In this way, government can play its role for achieving social objective. Hence, the modified objective function is given as follows:

\begin{equation*} U(\boldsymbol {\delta },\boldsymbol {d})=\alpha \sum _{i\in \mathcal {N}}\log (Z-\delta _{i}) + \beta \sum _{i\in \mathcal {N}}\log d_{i},\tag{6}\end{equation*}

where $\alpha =\alpha '\omega $ and $\beta =\beta '(1-\omega)$ with $\alpha '>0$ and $\beta '>0$ are incentives per unit of isolation and social distancing. In practice, $\alpha $ and $\beta $ can be monetary values for per unit of isolation and social distancing, respectively. In (6), one individual’s position affects the social distancing of others, and hence, the individuals have partially conflicting interest on the outcome of $U$. Therefore, the situation can be interpreted with the noncooperative game [36], [37].

A noncooperative game is a game that exhibit a competitive situation where each player (i.e., individual) needs to make choices independent of the other individuals, given the possible policies of the other individuals and their impact on the individual’s payoffs or utilities. Now, a noncooperative game in strategic form or a strategic game $\mathcal {G}$ is a triplet $\mathcal {G}=(\mathcal {N}, (\mathcal {S}_{i})_{i\in \mathcal {N}}, (u_{i})_{i\in \mathcal {N}})$ [40] for any time period where:

• $\mathcal {N}$ is a finite set of individuals, i.e., $\mathcal {N}=\{1,2,\cdots,N\}$,
• $\mathcal {S}_{i}$ is the set of available strategies for individual $i\in \mathcal {N}$,
• $u_{i}:\mathcal {S}\rightarrow \mathbb {R}$ is the payoff function of individual $i\in \mathcal {N}$, with $\mathcal {S}=\mathcal {S}_{1}\times \mathcal {S}_{2}\times..\times \mathcal {S}_{N}$.

Theoretically, there may be many different strategies, but in our case, we have considered two strategies, namely staying at home and moving outside (visit market, shop, train station, school/college etc.) for every individual. We use $\mathcal {S}_{i}=\{s_{i}^{h},s_{i}^{m}\}$ to represent the set of strategies for each individual $i$ where $s_{i}^{h}$ and $s_{i}^{m}$ indicate the strategies of staying at home and moving outside for individual $i\in \mathcal {N}$, respectively. The payoff or incentive function of any individual $i\in \mathcal {N}$ in a time period can be defined as follows:

\begin{align*} u_{i}(.) = \begin{cases} \alpha \log Z +\beta \log \tilde {d}_{i}, & \text {if strategy is}~s_{i}^{h}, \\ \alpha \log (Z-\delta _{i})+\beta \log d_{i}, & \text {if strategy is}~s_{i}^{m}. \end{cases}\tag{7}\end{align*}

where $\tilde {d}_{i}=\sum _{j\in \mathcal {N}_{i}}\sqrt {(X_{i}^{h}-X_{j})^{2}+(Y_{i}^{h}-Y_{j})^{2}}$.

The Nash equilibrium [38] is the most used solution concept for a noncooperative game. Formally, Nash equilibrium can be defined as follows [39]:

Definition 1: A pure strategy Nash equilibrium for a non-cooperative game $\mathcal {G}=(\mathcal {N}, (\mathcal {S}_{i})_{i\in \mathcal {N}}, (u_{i})_{i\in \mathcal {N}})$ is a strategy profile $\mathbf {s}^{*}\in \mathcal {S}$ where $u_{i}(s_{i}^{*},{\boldsymbol {s}}_{-i}^{*})\ge u_{i}(s_{i},{\boldsymbol {s}}_{-i}^{*}), \forall s_{i} \in \mathcal {S}_{i},\forall i\in \mathcal {N}$.

However, to find the Nash equilibrium, the following two definitions can be helpful.

Definition 2 *[40]*: *A strategy $s_{i}\in \mathcal {S}_{i}$ is the dominant strategy for individual $i\in \mathcal {N}$ if $u_{i}(s_{i},{s}_{-i})\ge u_{i}(s_{i}',{s}_{-i}), \forall s_{i}' \in \mathcal {S}$ and $\forall s_{-i} \in \mathcal {S}_{-i}$, where $\mathcal {S}_{-i}=\prod _{j\in \mathcal {N}, j\ne i}\mathcal {S}_{j}$ is the set of all strategy profiles for all individuals except $i$.*

Definition 3 *[40]*: A strategy profile $\mathbf {s}^{*}\in \mathcal {S}$ is the dominant strategy equilibrium if every elements $s_{i}^{*}$ of $\mathbf {s}^{*}$ is the dominant strategy of individual $i\in \mathcal {N}$.

Thus, if we can show that every individual of our game $\mathcal {G}$ has a strategy that gives better utility irrespective of other individuals strategies, then with the help of Definition 2 and 3, we can say that Proposition 1 is true.

Proposition 1: *$\mathcal {G}$ has a pure strategy Nash equilibrium when $\alpha >\beta $.*

Proof: Let us consider a 2-individuals simple matrix game as shown in Table 1 with the mentioned strategies. For simplicity, we consider Laplacian distance $\Delta $ that each individual can pass in any timestamp.

Thus, the utilities of $P_{1}$:

\begin{align*} u_{1}(s_{1}^{h},s_{2}^{h})=&\alpha \log Z + \beta \log d_{1}, \\ u_{1}(s_{1}^{h},s_{2}^{m})=&\alpha \log Z + \beta \log (d_{1}\pm \Delta), \\ u_{1}(s_{1}^{m},s_{2}^{h})=&\alpha \log (Z-\Delta) + \beta \log (d_{1}\pm \Delta), \\ u_{1}(s_{1}^{m},s_{2}^{m})=&\alpha \log (Z-\Delta) + \beta \log (d_{1}\pm 2\Delta), \tag{8}\end{align*}

where ± indicates the movement of individual to other individual and opposite direction, respectively. Now,

\begin{align*}&\hspace {-2pc}u_{1}(s_{1}^{h},s_{2}^{h})-u_{1}(s_{1}^{m},s_{2}^{h}) \\=&\alpha \log \left({\frac {Z}{Z-\Delta }}\right) + \beta \log \left({\frac {d_{1}}{d_{1}\pm \Delta }}\right), \\&\hspace {-2pc}u_{1}(s_{1}^{h},s_{2}^{m})-u_{1}(s_{1}^{m},s_{2}^{m}) \\=&\alpha \log \left({\frac {Z}{Z-\Delta }}\right) + \beta \log \left({\frac {d_{1}\pm \Delta }{d_{1}\pm 2\Delta }}\right). \tag{9}\end{align*}

As $\alpha >\beta $, so the following conditions hold from (9):

\begin{align*} u_{1}(s_{1}^{h},s_{2}^{h})-u_{1}(s_{1}^{m},s_{2}^{h})\ge&0, \\ u_{1}(s_{1}^{h},s_{2}^{m})-u_{1}(s_{1}^{m},s_{2}^{m})\ge&0, \tag{10}\end{align*}

Hence, rewriting (10), we get the followings:

\begin{align*} u_{1}(s_{1}^{h},s_{2}^{h})\ge&u_{1}(s_{1}^{m},s_{2}^{h}), \\ u_{1}(s_{1}^{h},s_{2}^{m})\ge&u_{1}(s_{1}^{m},s_{2}^{m}). \tag{11}\end{align*}

Thus, $s_{1}^{h}$ is the dominant strategy of $P_{1}$. Moreover, for the individual $P_{2}$, the utilities are as follows:

\begin{align*} u_{2}(s_{1}^{h},s_{2}^{h})=&\alpha \log Z + \beta \log d_{2}, \\ u_{2}(s_{1}^{m},s_{2}^{h})=&\alpha \log Z + \beta \log (d_{2}\pm \Delta), \\ u_{2}(s_{1}^{h},s_{2}^{m})=&\alpha \log (Z-\Delta) + \beta \log (d_{2}\pm \Delta), \\ u_{2}(s_{1}^{m},s_{2}^{m})=&\alpha \log (Z-\Delta) + \beta \log (d_{2}\pm 2\Delta). \tag{12}\end{align*}

Now,

\begin{align*}&\hspace {-2pc}u_{2}(s_{1}^{h},s_{2}^{h})-u_{2}(s_{1}^{h},s_{2}^{m}) \\=&\alpha \log \left({\frac {Z}{Z-\Delta }}\right) + \beta \log \left({\frac {d_{2}}{d_{2}\pm \Delta }}\right), \\&\hspace {-2pc}u_{2}(s_{1}^{m},s_{2}^{h})-u_{2}(s_{1}^{m},s_{2}^{m}) \\=&\alpha \log \left({\frac {Z}{Z-\Delta }}\right) + \beta \log \left({\frac {d_{2}\pm \Delta }{d_{2}\pm 2\Delta }}\right). \tag{13}\end{align*}

As $\alpha >\beta $, so the following conditions hold from (13):

\begin{align*} u_{2}(s_{1}^{h},s_{2}^{h})-u_{2}(s_{1}^{m},s_{2}^{m})\ge&0, \\ u_{2}(s_{1}^{m},s_{2}^{h})-u_{2}(s_{1}^{m},s_{2}^{m})\ge&0, \tag{14}\end{align*}

Hence, rewriting (14), we get the followings:

\begin{align*} u_{2}(s_{1}^{h},s_{2}^{h})\ge&u_{2}(s_{1}^{h},s_{2}^{m}), \\ u_{2}(s_{1}^{m},s_{2}^{h})\ge&u_{2}(s_{1}^{m},s_{2}^{m}). \tag{15}\end{align*}

Therefore, $s_{2}^{h}$ is the dominant strategy of individual $P_{2}$.

When there are $N$-individuals ($N>2$) in the game, every individual still has the same two strategies as in the case of a 2-individuals game. However, the dimension of the game matrix, which is actually representing the payoff of every individual for each strategy, will be changed from $2\times 2$ to $2^{N}$. The incentive of individual $i\in \mathcal {N}$ (takes strategy $s_{i}^{h}$, without considering others strategy), is given as follows:

\begin{equation*} u_{i}(s_{i}^{h}, {\dots }) = \alpha \log Z +\beta \log \tilde {d}_{i}.\tag{16}\end{equation*}

However, if the individual $i\in \mathcal {N}$ takes the strategy $s_{i}^{m}$, i.e., the individual visits some crowded place like market, shop, train station, school, or other location, then a person may come in close contact with many others. Thus, the incentive of individual $i\in \mathcal {N}$ with this strategy is given as follows:

\begin{equation*} u_{i}(s_{i}^{m}, {\dots }) = \alpha \log (Z-\delta _{i}) +\beta \log d_{i},\tag{17}\end{equation*}

where $\delta _{i}$ is calculated from (2) and $d_{i}$ is measured from (4) for that particular location. Moreover, $d_{i}< \tilde {d}_{i}$ as these places are crowded and individuals are in short distance with one another. Hence, $u_{i}(s_{i}^{h}, {\dots })>u_{i}(s_{i}^{m}, {\dots })$ as $Z>Z-\delta _{i}$ and $\tilde {d}_{i}>d_{i}$ for any individual $i\in \mathcal {N}$. That means, $s_{i}^{h}$ is the dominant strategy for individual $i\in \mathcal {N}$ irrespective of the strategies of other individuals in the game $\mathcal {G}$. Thus, there is a strategy profile $\boldsymbol {s}^{*}=\{s_{1}^{h},s_{2}^{h},\cdots,s_{N}^{h}\}\in \mathcal {S}$ where each element $s_{i}^{*}$ is a dominant strategy. Hence, by Definition 3, $\boldsymbol {s}^{*}$ is a dominant strategy equilibrium. Moreover, a dominant strategy equilibrium is always a Nash equilibrium [40]. Hence, the game $\mathcal {G}$ has always a pure strategy Nash equilibrium.

**TABLE 1:** Game Matrix for 2-Individuals

		P 2	
		$s_{2}^{h}$	$s_{2}^{m}$
P 1	$s_{1}^{h}$	$(u_{1}(s_{1}^{h},s_{2}^{h}),u_{2}(s_{1}^{h},s_{2}^{h}))$	$(u_{1}(s_{1}^{h},s_{2}^{m}),u_{2}(s_{1}^{h},s_{2}^{m}))$
	$s_{1}^{m}$	$(u_{1}(s_{1}^{m},s_{2}^{h}),u_{2}(s_{1}^{m},s_{2}^{h}))$	$(u_{1}(s_{1}^{m},s_{2}^{m}),u_{2}(s_{1}^{m},s_{2}^{m}))$

Thus, Nash equilibrium is the solution of the noncooperative game $\mathcal {G}$. In this equilibrium, no individual of $\mathcal {N}$ has the benefit of changing their strategy while others remain in their strategies. That means, the utility of each individual $i\in \mathcal {S}$ is maximized in this strategy, and hence ultimately maximize the utility of (6). In fact, incentivizing the social distancing mechanism is promoting social distancing to each individual. To this end, maximizing $U$ of (6) ultimately maximize the original objective function of (5).

Moreover, the Nash equilibrium point has a greater implication on controlling the spread of COVID-19 in the society. At the NE point, every individual stays at home, and that is the only NE point in our game environment. So, if someone gets affected by COVID-19, the individual will not go in contact with others. Similarly, an unaffected individual has no probability to come in contact with an affected individuals. Unfortunately, the family members have the chance to be affected if they don’t follow fair distance and health norms.

For calculating the utility of each player (i.e., individual) $i\in \mathcal {N}$ in case of any strategy, the positional information of $\mathcal {N}_{i}$ individuals who are in close proximity of $i\in \mathcal {N}$ are necessary. In order to obtain this information, each individual $i\in \mathcal {N}$ communicates with GPS satellite and it sends the information of $\mathcal {N}_{i}, \forall i\in \mathcal {N}$ individuals to the corresponding player $i\in \mathcal {N}$. Hence, GPS satellite needs to send $\sum _{i\in \mathcal {N}}|\mathcal {N}_{i}|=C|\mathcal {N}|$ information, where $C$ can be a fixed number to represent the close-proximity individuals and equal for every $i\in \mathcal {N}$, as a whole for $|\mathcal {N}|$ individuals. Thus, the complexity of the game is proportional to the number of players $|\mathcal {N}|$ of the game, and will not increase exponentially.

## V. Sustainability of Lockdown Policy With the System Model

The sustainability of the lockdown policy can be interpreted by using the outcome of the Nash equilibrium point that is achieved in the noncooperative game in Section IV.

The total amount of incentive a particular time period is presented in (6). In a particular day, we have $T_{s}=\frac {24\times 60}{T_{0}}$ time period where $T_{0}$ is the length of a time period in minutes. Thus, we can denote the incentive of a time stamp $t_{s}$ in a particular day $p$ as follows:

\begin{equation*} U_{p}^{t_{s}}(\boldsymbol {\delta },\boldsymbol {d})=\alpha \sum _{i\in \mathcal {N}}\log (Z-\delta _{i}) + \beta \sum _{i\in \mathcal {N}}\log d_{i}.\tag{18}\end{equation*}

Hence, the amount of resources/money that is necessary to incentivize individuals in a particular day, $p$ can be expressed as follows:

\begin{equation*} U_{p}= \sum _{t_{s}=1}^{T_{s}}U_{p}^{t_{s}}(\boldsymbol {\delta },\boldsymbol {d}).\tag{19}\end{equation*}

Now, if we are interested to find the sustainability of lockdown policy for a particular country till a certain number of days, denoted by $P$, we have to satisfy the following inequality:

\begin{equation*} \sum _{p=1}^{P}U_{p} \le R_{0} + \sum _{p=1}^{P}r_{p},\tag{20}\end{equation*}

where $R_{0}$ is the amount of resource/money of a particular country at the starting of lockdown policy that can be used as incentive and $r_{p}$ is the collected resources in a particular day, $p$, of the lockdown period. Here, $r_{p}$ includes governmental revenue and donation from different individuals, organizations and even countries. Moreover, the unit of $\alpha $, $\beta $, $R_{0}$ and $r_{p}$ are same.

If we assume for simplicity that $U_{p}$ and $r_{p}$ are same for every day and they are denoted by $\tilde {U}$ and $\tilde {r}$, respectively, then we can rewrite (20) as follows:

\begin{equation*} P\times \tilde {U} \le R_{0} + P\times \tilde {r}.\tag{21}\end{equation*}

Hence, if we are interested to find the upper limit of sustainable days for a particular country using lockdown policy, then we have the following equality:

\begin{equation*} P\times \tilde {U} = R_{0} + P\times \tilde {r}.\tag{22}\end{equation*}

Thus, by simplifying (22), we have the following:

\begin{equation*} P = \frac {R_{0}}{\tilde {U}-\tilde {r}}.\tag{23}\end{equation*}

Here, the sustainable days $P$ depends on $R_{0}$, $\tilde {U}$, and $\tilde {r}$. However, we cannot change $R_{0}$ but government can predict $\tilde {r}$. Moreover, depending on $R_{0}$ and $\tilde {r}$, government can formulate its policy to set $\alpha $ and $\beta $ so that individuals are encouraged to follow the lockdown policy. Alongside, we cannot continue lockdown policy infinitely based upon the limited total resources. Hence, the governments should formulate and update its lockdown policy based on the predicted sustainable capability to handle COVID-19, otherwise resource crisis will be a further bigger worldwide pandemic.

## VI. Numerical Analysis

In this section, we assess the proposed approach using numerical analyses. We consider an area of 1, 000 m $\times 1,000$ m for our analysis where individuals’ position are randomly distributed. The value of the principal simulation parameters are shown in the Table 2.

**TABLE 2:** Value of the Principal Simulation Parameters

Symbol	Value
$N$	{500, 1000, 1500, 2000}
$\alpha $	3.0
$\beta $	1.0
$\omega $	[0, 1]
$Z$	1400
$R_{0}$	$\{5\times 10^{23},5.5\times 10^{23},6\times 10^{23},6.5\times 10^{23},7\times 10^{23}\}$
$\tilde {r}$	$\{\tilde {U}\times 0.10, \tilde {U}\times 0.20, \tilde {U}\times 0.30, \tilde {U}\times 0.40, \tilde {U}\times 0.50\}$

**FIGURE 2.**
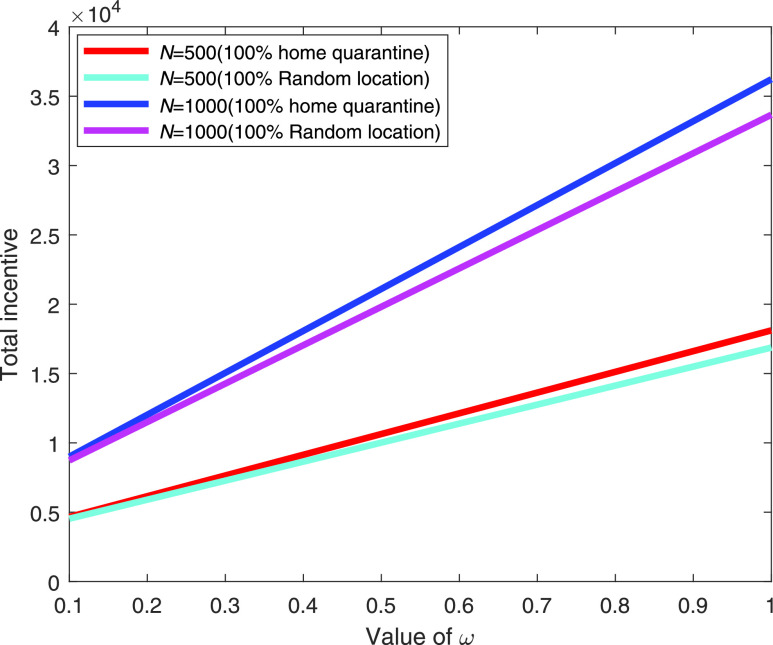
Comparison of incentive (in log scale) for varying value of $\omega $.

Figure 2 illustrates a comparison between home isolation (stay at home) and random location in the considered area for a varying value of $\omega $. In this figure, we consider two cases of $N = 500$ and $N=1,000$. In both the cases, home isolation (quarantine) is beneficial over staying in random location and the differences between two approaches are increased with the increasing value of $\omega $. Moreover, the difference of payoffs between two approaches are increased with the increasing value of $\omega $ as the more importance are given in home isolation.

**FIGURE 3.**
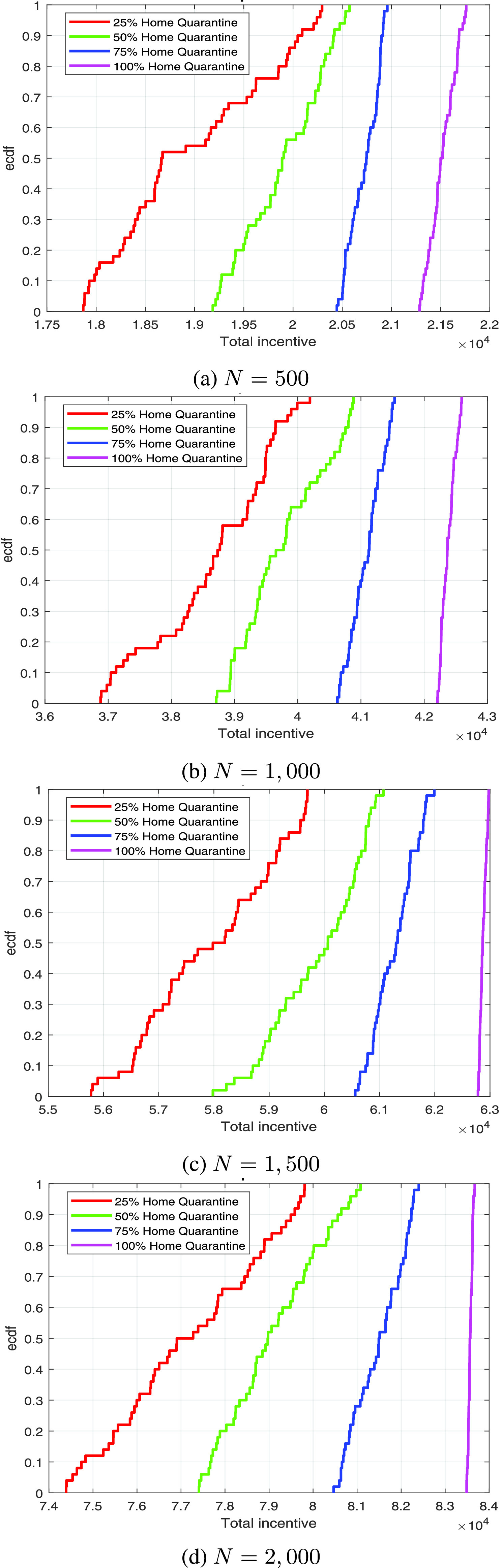
Ecdf of incentives (in log scale) for different value of $N$ with $\alpha =3.0$ and $\beta =1.0$ using 50 runs.

Figure 3 shows the empirical cumulative distribution function (ecdf) of incentives for different numbers of individuals. The figure revels that the incentive values increase with the increasing number of home quarantine individuals in all the four cases. Figure 3a exhibits that the incentives are below 19, 000, and 20, 000 for 50%, and 48% sure, respectively, for 25% and 50% home quarantine cases whereas the incentives are 90% sure in between 20, 500 and 21, 000 for 75% home isolation case. Further, the same values are at least 21, 500 for 50% sure in case of full home isolation. Figure 3b depicts that the incentive of being below 38, 000 is 40% sure for 25% home isolation case, however, the same values of being above 40, 000, and 41, 000 are 40%, and 60%, sure, respective, for 50%, and 75% cases. Moreover, for 100% home isolation case, the values are in between 42, 000 to 43, 000 for sure. The incentives for 25%, 50%, 75%, and 100% home isolation cases are above 57, 000, 59, 000, 61, 000, and 63, 000, respectively, with probability 0.60, 0.65, 0.65, and 0.80, respectively, as shown in Figure 3c. Additionally, the same values are at least 77, 000, 79, 000, 81, 000, and 83, 500 with 0.50, 0.50, 0.72, and 1.00 probabilities, respectively, which is presented in Figure 3d.

**FIGURE 4.**
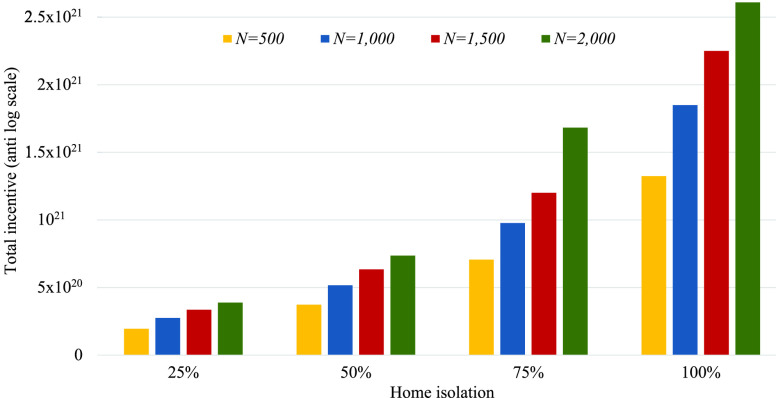
Total incentive (average of 50 runs) for varying percentage of home isolation individuals when $\alpha =3.0$ and $\beta =1.0$.

The total incentive (averaging of 50 runs) for varying percentage of home isolation individuals with different sample size are shown in Figure 4. From this figure, we observe that the total payoff increases with increasing number of home isolation individuals for all considered cases. The incentives are 578%, 571%, 571%, and 571% better from home quarantine of 25% to 100% for $N=500$, $N=1,000$, $N=1,500$, and $N=2,000$, respectively. Moreover, for a particular percentage of home isolation, the total incentive is related with the sample size. In case of 50% individuals in the home isolation, the incentive for $N=2,000$ is 97.08%, 42.50%, and 15.96% more than that of $N=500$, $N=1,000$, and $N=1,500$, respectively.

**FIGURE 5.**
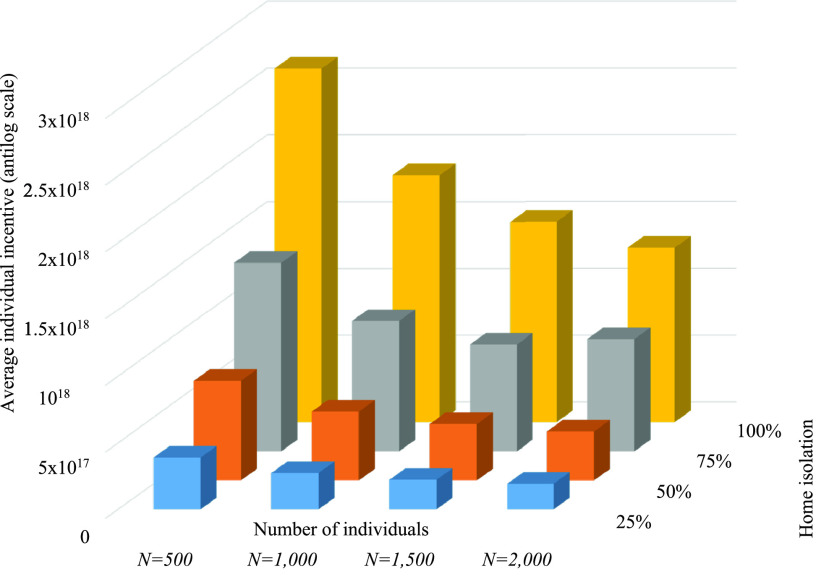
Average individual incentive for varying percentage of home quarantine individuals when $\alpha =3.0$ and $\beta =1.0$.

Figure 5 shows the average individual payoff for varying parentage of home isolation individuals for different scenarios. The figure exhibits that the average individual incentive increases with an increasing percentage of home isolation as the deviation $\boldsymbol {\delta }$ decreases and hence, the value of home isolation incentive increases. For $N=500$, the incentive of 100% home isolation is 85.25% more than that of 25% home isolation. Moreover, in a particular percentage of home isolation, the incentive decreases with an increasing number of considered individuals as the social distancing decreases due to the more number of individuals. In case of 50% home isolation, the individual incentive for $N=500$ is 102.96% more than that of $N=2,000$.

**FIGURE 6.**
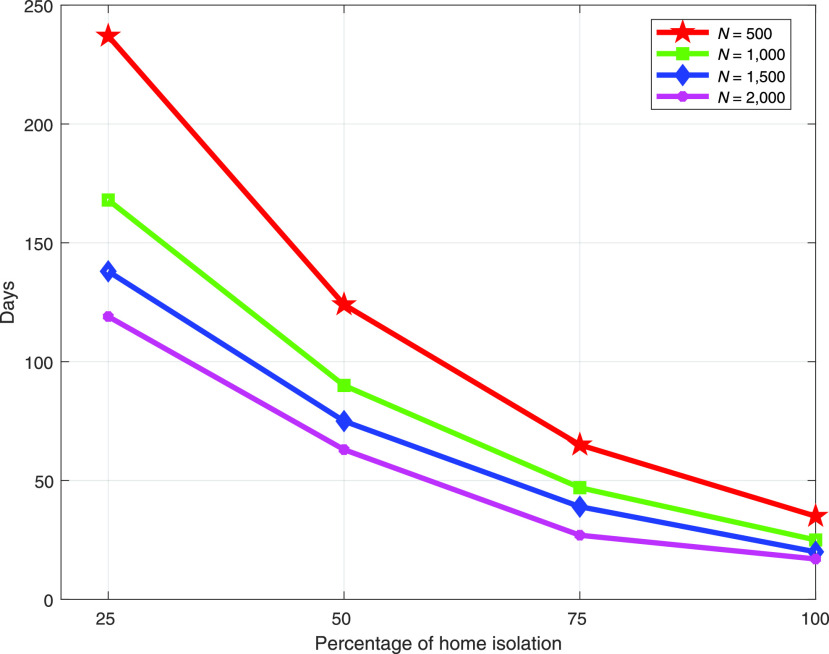
Maximum possible lockdown period with varying number of individuals when $R_{0}=5\times 10^{23}$, ${\tilde {r}=0.10\times \tilde {U}}$, and using total incentive shown in Figure 4.

**FIGURE 7.**
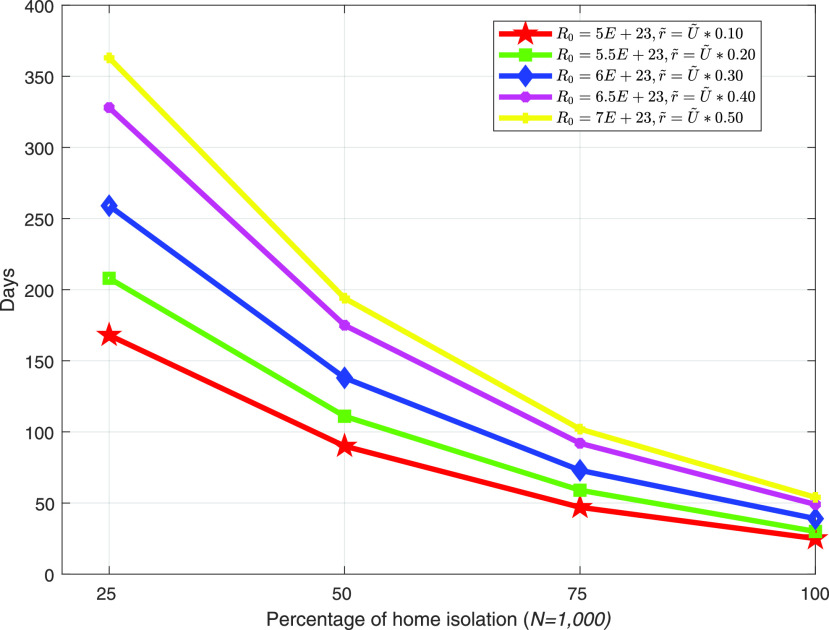
Maximum possible lockdown period with varying $R_{0}$ and $\tilde {r}$ with total payoff shown in Figure 4.

Figure 6 shows the maximum possible lockdown period for a varying number of individuals within a fixed amount of resource $R_{0}$. The figure reveals that with the increasing percentage of home isolation individuals, the maximum lockdown period significantly decreases for all considered cases. The reason behind this is that the more individuals are in home isolation, the more it is necessary to pay the incentives. With a fixed amount of resources, a country with less individuals can survive a longer lockdown period. With more percentages of home isolation individuals, the number of lockdown period is less, and possible of spreading of COVID-19 is also less. Therefore, the governments can consider a trade-off between increasing expenditure as a incentive and lockdown period. For 1, 000 individuals, the maximum possible lockdown period for varying amount of $R_{0}$ and $\tilde {r}$ is presented in Figure 7. The figure also illustrates that with the increasing percentages of home isolation individuals, the continuity of the lockdown period reduces for every scenarios. However, for a particular percentage of home isolation individuals where total number of individuals are fixed, a country can continue higher lockdown period who has more am amount of resources, $R_{0}$. Additionally, $\tilde {r}$ also play an important role to continue the lockdown period.

## VII. Discussion and Limitation of the Study

The limitations of the current study are summarized as follows:

• The governments are required to formulate and update their lockdown policy over time while mitigating the financial impact of COVID-19. From equations (20) – (23) we observe that such a policy update requires significant financial planning and prediction of the government revenue over a timespan while considering the socio-economic conditions of the population. In other words, the financial condition of the population has a considerable impact on the success of any distributed (i.e., less government control) or centralized (i.e., strict government control) lockdown policies.
• The proposed noncooperative game solution provides an analytical approach to attain the solution that keeps the total deviation from home $\delta $ by an individual from a vast population to the minimum for reducing the infection risk of COVID-19. Besides, such a game solution provides a tractable evaluation for the sustainability of the lockdown policy. However, like the other mathematical models of epidemic diseases (i.e., SI, SIR, SIRS, SEIR), incorporating the probabilities of infection and recovery for COVID-19 in the noncooperative game setting is still an open research question and requires further investigation.

To this end, in the future, we will further study a stochastic game setting by incorporating epidemic cases as a dynamics of Markovian for capturing the uncertain behavior of pandemic. In particular, the policy for the government-controlled epidemic models will be analyzed by the multi-agent noncooperative reinforcement learning for coping with an unknown epidemic environment. Therefore, the government/institute will capable of taking a proactive policy measurement for enhancing the sustainability of any kind of epidemic.

## VIII. Conclusion

In this article, we have introduced a mathematical model for controlling the outbreak of COVID-19 by augmenting isolation and social distancing features of individuals. We have solved the utility maximization problem by using a noncooperative game. Here, we have proved that staying home (home isolation) is the best strategy of every individual and there is a Nash equilibrium of the game. By applying the proposed model, we have also analyzed the sustainability period of a country with a lockdown policy. Finally, we have performed a detailed numerical analysis of the proposed model to control the outbreak of the COVID-19. In future, we will further study and compare with extended cases such as centralized and different game-theoretic models. In particular, an extensive analysis between the government-controlled spread or individual controlled spread under more diverse epidemic models.
